# Challenges in physician-patient communication for optimal management of systemic sclerosis-associated interstitial lung disease: a discourse analysis

**DOI:** 10.1007/s10067-020-05063-x

**Published:** 2020-04-13

**Authors:** Christopher P. Denton, Bee Laird, Lizette Moros, Jose Luis Luna Flores

**Affiliations:** 1 Division of Medicine, Department of Inflammation, Centre for Rheumatology and Connective Tissue Diseases, Royal Free and University College Medical School, University College London, London, UK; 2 The Research Unit, Hove, UK; 3 Boehringer Ingelheim International GmbH, Ingelheim am Rhein, Germany

**Keywords:** Interstitial lung disease, Patient understanding, Physician-patient communication, Systemic sclerosis

## Abstract

**Introduction:**

Systemic sclerosis (SSc) is a rare, potentially life-threatening condition. The prognosis is difficult to predict, and treatment is complex. This can be difficult to understand or explain, posing challenges for effective physician-patient communication. Our study assessed communication between physicians and patients with systemic sclerosis-associated interstitial lung disease (SSc-ILD) to identify information gaps and needs.

**Methods:**

Twenty-three 20-min consultations between physicians (rheumatologists, pulmonologists) and patients (19 real, 4 actors) with diagnosed SSc-ILD across 6 countries were observed and recorded. Interactional sociolinguistic discourse analysis was used to understand the pattern and meaning of communication, whether the needs of both participants were met, and the level of understanding between participants.

**Results:**

In most consultations, patients were given little opportunity to explain their concerns or ask questions. Physicians used plain language but would revert to medical terminology for complex issues. Patients would also use medical terminology, despite not fully understanding the terms, which led to some physicians mistakenly believing that patients had a better understanding than they did. Differences in cognitive models between physicians and patients were often responsible for misunderstandings. However, during effective consultations, patients were invited to tell their story, and physicians used techniques to check and demonstrate understanding, express empathy and build rapport.

**Conclusions:**

Communication challenges between physicians and patients limit joint understanding of SSc-ILD and may result in both parties misunderstanding important information and patients being less aware of self-help management approaches. Strategies should be developed with physicians to facilitate effective communication and increase patient understanding and support.

**Table:** 

**Key Points** *• Physicians and patients have different explanatory models for SSc-ILD.* *• The differences between the physician-constructed model of SSc-ILD and the personal model patients construct often result in misunderstandings. * *• Many patients with SSc have learned the medical terminology for their condition, but their often limited understanding may lead to missed opportunities for physicians to provide clear explanations and correct misperceptions.* *• The style of consultation determines the information patients share and the degree to which they feel supported by, and confident in, their physicians’ care. *	

## Introduction

Systemic sclerosis (SSc) is a rare but severe disease in terms of the burden of symptoms on the patient, the unpredictability of prognosis and the risk of premature death [1–3]. SSc typically affects multiple organs, including the lungs, where interstitial lung disease (ILD) and pulmonary arterial hypertension (PAH) may develop. ILD (along with PAH) is now the leading cause of SSc-associated mortality [4].

In addition to the symptoms and functional limitations associated with SSc, there are other important consequences of the experience and management of the disease that negatively impact on patients. These include feelings that healthcare professionals (HCPs) have little insight into their condition and needs [5]. Studies from other chronic, unpredictably progressive diseases have illustrated the scale and complexity of communication problems that can exist between healthcare professionals and patients. An impersonal or overly clinical approach, “one-way” interactions, perceived indifference to a patient’s situation and emotions, as well as a lack of empathy, support and respect can all contribute to make the consultations a negative experience. These negative consultations can lead to patients feeling alienated and excluded from decisions about their own care [6–9].

The depth and complexity of information that patients’ desire varies widely; while some wish to be fully informed of their prognosis, others prefer less information. Ideally, the amount of information provided should be personalised, communicated in the right way, at the right time and with the appropriate people present [10]. Studies have shown negative treatment outcomes occur because of miscommunication between the physician and the patient. In contrast, patients may perceive a good or positive consultation with a physician as indicative of the quality of care they are likely to receive [11]. This has been shown in heart failure (HF) studies where empathetic conversations with physicians improved the patient’s knowledge about the disease, enabling them to better judge when they needed to seek medical help or better manage their illness [8, 12]. In addition, feeling that they have been listened to and respected by their HCP as well as being in an active partnership can enhance patients’ self-care [6].

Patients with SSc-ILD have complex needs in relation to clinical consultations and decision-making. As SSc is a rare condition, patients may perceive it as a new, mysterious illness and therefore difficult to explain [13]. The relationship between SSc and ILD may be challenging for some patients to understand [14]. The most common information needs identified by patients when asked to consider the management of this condition were understanding test results, why tests are needed, treatment options and knowing when to seek medical attention. The aim of this study was to explore the communication between physicians and patients with SSc-ILD to identify the areas of mutual understanding, the information gaps and the patient needs.

## Materials and methods

This was a qualitative observational study designed to capture conversations and interactions between physicians and patients for critical discourse analysis. To ensure the natural language and intercourse between physicians and patients was captured, 20-min consultations between physicians (rheumatologists, pulmonologists) and patients with SSc-ILD in Germany, Italy, Spain, the UK and the USA were observed. In Japan, where it was not possible to include real patients, consultations were observed between real physicians and actor patients. Consultations were directly observed by an independent researcher who took no part in the consultation. These consultations took place either face to face, in a neutral setting that was hired for the purposes of the study, or by Skype call. All conversations were audio recorded and conducted in the relevant native language. In the consultation, the interactions and discussions were controlled by the physician and patient. There was no script or discussion guide.

These consultations were followed immediately with an individual in-depth interview with each participant. In each country the interviews were conducted by professional, experienced, trained qualitative interviewers who were native speakers. During the in-depth interviews, visualisation and visual metaphors were elicited to clarify the meaning of important but nebulous terms and capture the emotional meaning of SSc. Cognitive language maps formed the basis of the discussion about what was said and understood and what was misunderstood or misinterpreted, enabling the dissonance between physicians and patients to be identified and characterised.

The procedures followed in this study were in accordance with the Declaration of Helsinki. All participants provided written informed consent before taking part in the study, and all study data were held according to European Union (EU) data protection laws. The research outline was discussed with the Reading Independent Ethics Committee (UK), who advised that no ethics committee review was required. Additionally, and per the European Pharmaceutical Market Research Association (EphMRA) Code of Conduct 2019, this research was classified as market research and therefore does not require ethics committee approval (includes Brazil, Canada, Denmark, Finland, France, Germany, Greece, Italy, Japan, Mexico, the Netherlands, Norway, Poland, Russia, South Korea, Spain, Sweden, Turkey, the UK and the USA). This report conforms to the Standards for Reporting Qualitative Research (SRQR) guidelines [15].

### Participants

All participants were recruited using researchers based in the respective countries. Patients were recruited by physicians who verified their diagnosis of SSc-ILD.

Specialist physicians were established rheumatologists or pulmonologists with relevant specialist experience. They were required to be regularly managing patients with SSc-ILD, conducting at least one consultation every 2 months and to see four or more patients with SSc-ILD per year. Additionally, they were required to spend at least 75% of their time directly caring for patients.

Patients with SSc-ILD were diagnosed with lung involvement by a medical specialist using computer tomography (CT). Patients were required to have ILD that limited their ability to conduct moderate- or vigorous-intensity physical activity. This study included patients with wide range of disease severities and the involvement of other organs.

None of the physicians or patients were known to one another.

### Analyses

All recordings were transcribed in their native language and translated into UK English by specialist medical translators. Data from each participant was anonymised for the analysis using a numerical code (e.g. UK DR 1).

Audio recordings from each consultation were analysed using linguistic techniques based on critical discourse analysis. Specifically, three key domains were explored: (1) the pattern and meaning of communication; (2) whether the needs of both participants were met; and (3) the level of understanding between participants. More specifically, the dynamics and tone of each participant’s contribution to the conversation and the use of specific language were explored.

Each transcript was independently analysed by two experienced qualitative analysts, and their coding frames and analyses were compared. Where there was discrepancy, the analysts discussed and agreed a modified analysis. There were internal triangulation checks built into the data; both physicians and patients were asked in the interview what their understanding and experience of the consultation had been, and patients were given an opportunity to tell their unprompted stories of their SSc-ILD (which were compared with the stories they told/were able to tell in the consultation).

## Results

### The observed consultations

This study included 23 mock consultations between 10 rheumatologists, 8 pulmonologists, 1 dermatologist and 1 general practitioner (GP) specialising in SSc and 19 patients with SSc-ILD (Germany, 5; Italy, 4; Spain, 2; UK, 4; USA, 4) and 4 actor patients in Japan (Table 1). These consultations were conducted and observed between November 2016 and January 2017. The size of the sample was pre-determined by the rarity of the disease, and patients were selected to represent a range of severities of SSc-ILD.

**Table 1:** Overview of the research sample

	Mock consultations		Face to face interviews	
	Patients (*n*)	Physicians (*n*)	Patients (*n*)	Physicians (*n*)
Germany	5	2 rheumatologists 1 pulmonologist 1 dermatologist 1 general practitioner (GP)	5	2 rheumatologists 1 pulmonologist 1 dermatologist 1 GP
Italy	4	3 rheumatologists 1 pulmonologist	4	3 rheumatologists 1 pulmonologist
Spain	2	2 pulmonologists	2	2 pulmonologists
UK	4	1 rheumatologist	4	0^a^
USA	4	2 rheumatologists 2 pulmonologists	4	2 rheumatologists 2 pulmonologists
Japan	4^b^	2 rheumatologists 2 pulmonologists	0^b^	2 rheumatologists 2 pulmonologists

^a^No physician interviews were conducted in the UK. The same rheumatologist “consulted” all 4 patients

^b^As patients were not available, actors were used to play the part of patients with SSc-ILD in Japan

The length of specialist experience amongst physicians was between 3 and 30 years, and the physicians treated an average of 32 patients with SSc-ILD every year. Patients were aged 34 to 79 years, with a disease duration of 1 to 29 years from diagnosis, and all had multiple organ involvement including the skin, hands, feet, eyes, gastrointestinal system and joints.

### Analysis of the communication challenges

The analysis found that factors inhibiting effective physician-patient communication fell into three main categories: (a) consultation style, (b) meaning of language and the disconnect with use and (c) explanatory models—differences between physician and patient understanding of the disease.

### Consultation style

There were three different styles of consultation:

1. An easy, flowing dialogue between patient and physician, with the physician showing good listening skills, allowing the patient to talk about the non-clinical impact of their disease as well as just the clinical aspects, and using techniques to check and demonstrate understanding, express empathy and build rapport (see Table 2 for examples of techniques used by physicians within the consultations).
2. A “Q&A” session where the physician has a list of “screening questions” to ask the patient, and they roll from one question to the next, still in a flowing discussion but with a clearer, more clinical direction. Physicians solicited the patient’s story but then quickly diverted the course of the conversation to extract clinical information from the patient. In addition, physicians sometimes asked more than one question at a time, which confused the patient and gave them the impression that the physician was in a hurry. In a few cases, physicians re-ordered the sequence of the patient’s story as the consultation proceeded to fit their knowledge of SSc.
3. A consultation where the physician does most of the talking. Physicians use the opportunity to educate rather than to uncover the patient’s story. Occasionally, physicians asked a series of questions and then answered some of these questions for the patient without waiting for the patient’s response.

**Table 2:** Direct quotes from physicians and patients made during and immediately after the consultation

Empathy	Patient: “Yes, I kind of get, you know, all the symptoms possible.” Physician: A little overwhelming, I’m sure, right?”
Rapport building	Physician: “Do you live by yourself or with family?” Patient: “I live with my daughter, my 17-year-old.” Physician: “That’s a headache. I’m sorry, I have kids too.” Patient: “Oh my God. I agree, totally. I just cannot wait until she’s off to college. I mean, I need a vacation.” Physician: “Okay, uh-oh. I will not tell her you said that.”
Consultation pattern (patient quotes)	“He was talking non-stop.” “He had his things to say. He did not stop to listen to what I was saying.” “Sometimes I see him writing in [sic] the computer and he asks, ‘And how have you been?’ And he keeps writing.”
Use and meaning of language during the consultation (patient quotes)	“If [doctors] use technical terms, it’s beyond me, but a lot of them do that… It used to be like that—I went to lots of doctors, and they told me all sorts of things, but I did not know what they meant, but then I found out for myself.” “Of course [the doctor] assumed a lot of knowledge in me, about scleroderma... and also the lung involvement, he also used many specialist terms like fibrosis, lung fibrosis, of course he also wanted a lot of specialist knowledge from me.”
Understanding of SSc-ILD (patient quotes)	“[The doctor] said it is a congenital disease, it is not because of something I had done or had not done to develop this disease; she said that the body itself rejects those cells or something like that…” “My body produces too much collagen, it’s hard to explain. There are deposits and that is what causes everything to swell, especially the oesophagus, it gets narrower. [The lungs] harden because of the collagen deposits, they get swollen, and this stops them from working properly.”

In many consultations, patients were given little opportunity to explain their concerns or ask questions. The interviewing tactics employed by many physicians during the consultations were often perceived as negative by the patients.

### Meaning of language and the disconnect with use

When observed in this study, physicians initially used plain (lay) language in their explanations to patients but then often reverted to medical terminology when describing complex issues, for example, if they were asked to explain what SSc is and how the disease and its symptoms fit together. Physician language tended to be unemotional and matter of fact and lacking in imagery and metaphor. When metaphors were used by physicians, these helped to give patients a clearer understanding of the issue being discussed. For example, when talking about SSc, one physician described the muscle affected by SSc as being like “wood” and another described patients’ lungs as being like “darker alleys, with constriction and small spaces, and instead of a balloon that you can push on, there are just more rigid walls, with some very narrow areas”.

Patients’ language tended to be functional and factual, unless the physician demonstrated emotional empathy, which elicited more emotional language from the patient. Overall, the patient tone was physician led and was influenced by the questions the physician asked and the way the physician asked these questions. The focus of the discussion was on providing information the physician was seeking rather than allowing the patient to share their whole story. During the consultation, patients often used medical terms but had limited understanding of the meaning. This sometimes led to the physician thinking the patient had a better understanding of issues around SSc than was the case and providing further information in very medical terms which patients struggled to understand. Table 3 provides examples of patients’ use of medical terminology which led physicians to believe they had a better understanding of their SSc-ILD than they actually did. There was also a disconnect in the terminology used around the disease, with physicians using the words “systemic sclerosis” while patients preferred “scleroderma”.

**Table 3:** Examples of patients’ use of medical terminology leading physicians to think they have a better understanding of their SSc-ILD than they actually have

Example 1 Language the patient used in the consultation: • CellCept mycophenolate • CAT scan • Early mild fibrosis • Discoid lupus • Hypothyroidism • Pernicious anaemia • Raynaud’s syndrome • Vitiligo Physician feedback from consultation: “She seems knowledgeable. She seemed informed about the tests that were done, the reasons that things were done and the reason things were started and stopped”. This is the patient’s understanding/explanation for her scleroderma: “What’s going on inside is that some of my cells have gone berserk. They’re supposed to do certain things: They’re supposed to keep my skin smooth. They’re supposed to oil my skin. They’re supposed to keep it soft and moist. They’re supposed to protect it from the outside environment. Those cells have lost their mind and they are now attacking myself, as if I am an enemy”. Example 2 What the patient said in the consultation: “I have GERD and as far as the symptoms of that go, the only thing I think that is troublesome for me is malnutrition. I have Sjögren’s so I have the dry eyes and mouth. Scleroderma’s my primary and Raynaud’s and Sjögren’s were definitely secondary. I’m taking CellCept and methotrexate”. Physician feedback from the consultation: “I think my role as a physician is to educate. I mean, doctor means teacher. So just educating her about her disease—which she seemed very knowledgeable about”. This is the patient’s understanding/explanation of her scleroderma: “I do not want to blame my pregnancy for triggering something. It’s so weird how I do not know if I want to say my pregnancy definitely triggered it or brought it out more—but they cannot—oh, of course they can say what it was really [the cause of her scleroderma], but I also do believe that it’s probably from both my parents’ genetics. I think that’s another reason why I probably cannot gain weight, because the skin is so tight. I think it’s the overproduction of collagen is affecting the tissue. I mean I lost my muscle. I do not have a lot of that muscle left”.	

### Explanatory models—differences between physician and patient understanding of the disease

Explanatory models, first described by the physician and medical anthropologist Arthur Kleinman, are the cognitive models we build to explain and make sense of illness. Explanatory models are not diagnostic tools but can provide physicians with an idea of how patients experience and interpret their conditions [16].

Physicians have their own explanatory model that is largely based on the medical model/understanding of disease, reflecting their professional knowledge and experience; this usually determines the questions physicians ask and how they interpret the answer patients give them. Patients’ explanatory models are built on personal knowledge, beliefs and experiences of illness and determine how patients make sense of the disease and treatment information provided by their physicians. As a result, there is often a discrepancy between patients’ and physicians’ explanatory models that explains why there are misunderstandings and why some patients reject medication or refuse to comply with a prescribed therapy.

**Fig. 1:**
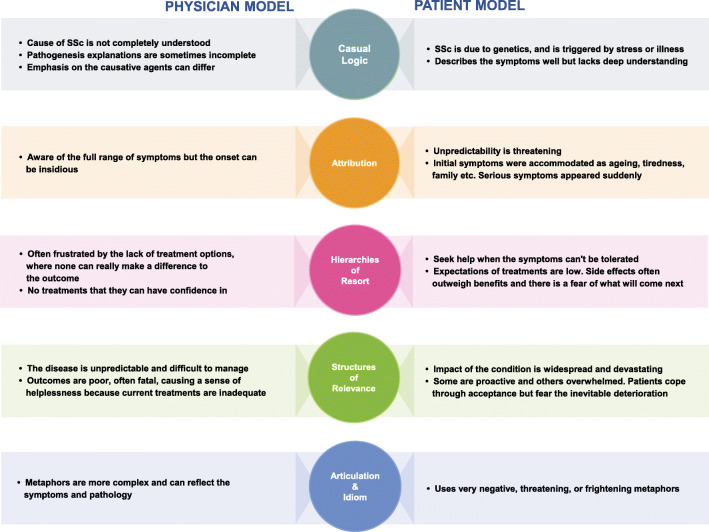
Physician and patient explanatory models in SSc

Physicians and patients have different ways of understanding SSc-ILD, based on their knowledge, experiences, expectations and personal beliefs (Fig. 1). These determined what information the patient shared and how they interpreted information provided by their physician. Patients’ understanding of what SSc-ILD is and what causes it often differed from the medical model of the disease. Beliefs about causes, symptoms and transmissibility were often only partly correct or based upon misconceptions. Even patients who had been diagnosed for many years had a limited understanding of the disease process and how their symptoms fitted together. Differences in the explanatory models used by physicians and patients were responsible for these misunderstandings. Most patients thought the cause of SSc and SSc-ILD was genetic and that it was triggered by stress or illness. Many of the symptoms were described in detail by patients, with some references made to connective tissue and fibrosis. However, there was generally a superficial or self-constructed understanding of how it all fitted together.

### Similarities and differences between countries

The range of consultation styles was observed across all of the countries with the exception of Japan, where the consultations all followed the pattern of a “Q&A” session, with the physician directing the consultation using more technical medical language. Patients’ use of language and terms to describe their SSc-ILD and symptoms was universal; the same or similar terms were used across all countries, and there were no differences between countries in the terms used by physicians.

## Discussion

This study identified three key domains of physician-patient communication that can impact positively or negatively on the SSc-ILD patient’s experience of clinical consultations. Physicians who controlled the consultation and did not allow patients time to ask their own questions contributed to a negative experience. The language used by physicians significantly influenced how patients felt about the consultations; a lack of warmth and empathy created distance between participants, while the use of metaphors encouraged more patient engagement. Notably, despite using medical terminology, patients often did not know what it meant. Physicians therefore assumed patients were more knowledgeable than they were and explained things in a complex manner that patients failed to understand. Using empathy and building rapport was welcomed by patients and led to a more positive consultation experience.

Patients with SSc need to feel confident about their interactions with physicians, and this depends on the physician’s medical and interpersonal skills, including their ability to individualise the relationship [13]. Findings from this study are consistent with other studies of physician-patient communication within consultations and their impact on emotional well-being. Both a lack of empathy from HCPs and their unwillingness to discuss aspects not specifically related to SSc have previously been cited by patients as a great source of emotional distress [17]. Additionally, patient’s dissatisfaction has been shown to relate to the absence of a holistic approach to care and due to a lack of tact by physicians, who they felt had little interest in their suffering [13]. Literature on chronic illness generally supports rapport building as a crucial step in fostering trust between physicians and patients [18]. Not being “present” and attentive to patient needs can lead to a focus on issues that are not important to the patient. Allowing patients a time to tell their story if they need to may encourage a more positive experience. The degree of patient participation in consultations has been shown to be specifically related to physicians’ verbal and non-verbal encouragement and reflection of facts and emotions [19]. In patients with HF, negative feelings following conversations with certain physicians resulted in wariness about future consultations and a lack of confidence in the physician [20]. As in this study in SSc-ILD, other studies have shown that patients with HF did not get the opportunity to ask questions, often because they perceived the physician to be too busy and because they did not feel empowered to start this kind of discussion [21]. However, a physician carefully exploring and respecting a patient’s hopes, fears and goals can ensure that the consultation is a positive experience for the patient [22].

The communication experience between the physician and patient can have both direct and indirect influences on outcomes. In patients with HF, face-to-face conversations with HCPs rather than by telephone have been shown to be associated with a decrease in hospital readmissions [23]. Health outcomes are also affected indirectly, including patient-enhanced satisfaction with care and empowerment, improved motivation regarding adherence and self-care, and increased knowledge and self-efficacy [24, 25].

Forming a therapeutic partnership with the physician is vital to many patients who have serious and chronic illnesses. This can facilitate the physician’s understanding of the patient’s wishes and beliefs while also identifying any misconceptions about the illness and its management [24]. It has been shown that in patients with bowel or breast cancer, a physician’s empathy is associated with shared decision-making and reduces any regret the patient might have about the treatment choices they have made [26]. Conversely, less participation than the patient wanted was associated with a higher level of regret regarding treatment decisions. Similar findings have been reported in patients with multiple sclerosis (MS) in terms of their choice of disease-modifying therapy [27]. Negative communication experiences have led to some patients with MS perceiving their neurologists as having little interest in their day-to-day ongoing difficulties and stress [28, 29].

Language, or rather the use of medical terminology during patient consultations, is a common problem throughout medicine. In a study of rheumatology consultations, although almost 80% of physicians did not explain the medical words they used, patient responses did not indicate whether they had understood [30]. As in our study, many patients began using the same words and phrases for themselves in the discussion. Inappropriate language can substantially impact patients. For example, if a patient with HF is told he is failing drug therapy, he will most likely blame himself, even if this was not the physician's intention [22].

Poor understanding of their disease can lead to uncertainty, anxiety, fear and even disappointment for patients, including those with SSc-ILD [14, 31]. Indeed, it has been shown that patients with SSc were fearful when their symptoms progressed but remained unexplained by HCPs [32]. One study highlighted SSc patients’ misunderstanding of the disease that are similar to those in our study and included personal views about the causes of SSc [13].

There are many key positive and negative factors identified in this study that could improve future consultations between physicians and patients. Although patients expect physicians to be knowledgeable about their condition, they also need them to be human and attentive. Honest and empathetic two-way communication between physicians and patients is critical and can help to mitigate potential feelings of devastation when receiving prognoses in SSc. Recognising each patient’s experience of their disease as unique is paramount to successful communication, with information provided according to the individual’s needs and preferences. Checking a patient’s understanding before making decisions about investigations and management will help to engender a feeling of partnership and build rapport and trust. Patients need to feel sufficiently confident to speak up when they do not understand what the physician is saying, when they feel they are not being listened to or their personal priorities are not being met.

There are a few limitations to this study; these data provide country-level results, and the small sample sizes mean the results cannot be extrapolated to the wider population. One of the inclusion criteria was the limited ability to conduct moderate physical activity. Thus, all participants had relevant symptoms. There is evidence that the quality of life of patients with ILD is more related to their symptoms than the severity of lung impairment. Therefore, the participants may have represented patients with ILD who were more concerned (and aware) about their health status and more prone to listen to physicians than patients with asymptomatic disease. Additionally, a mock consultation with an unknown physician could influence the conduct of the conversation relative to conversations between a physician and their patients. Moreover, physicians and patients may behave differently when being observed by a third person or when participating in a study. However, even within this small sample, similarities in the range of consultation styles and in patient and physician language were notable across countries. There are also confounding factors like educational status, severity of disease and coexisting psychiatric illnesses like depression that can influence the outcome of the physician-patient interaction. To some extent, these were represented in the range of patients included in this research, but patients were not purposively recruited to represent these possible confounders, and their influence was not specifically examined in the analysis.

The findings of the present study suggest that the communication challenges in SSc-ILD seen between physicians and patients can limit both of their understanding of the condition, thus hindering effective management and self-care. Techniques to check and demonstrate understanding, expressing empathy and using metaphors to build rapport can aid patient understanding. Physician awareness that patients may have very different cognitive models may help to identify and correct misperceptions, understand key patient concerns, promote patient adherence and, using this greater understanding, optimise patient-physician communication. These are important factors that can be used to ensure the success of the consultation. Consistent strategies therefore need to be codeveloped and implemented in order to facilitate effective communication and provide support, increase patients’ understanding of SSc-ILD and ultimately improve the patient experience.
